# Report of clinical bone age assessment using deep learning for an Asian population in Taiwan

**DOI:** 10.37796/2211-8039.1256

**Published:** 2021-09-01

**Authors:** Chi Fung Cheng, Eddie Tzung-Chi Huang, Jung-Tsung Kuo, Ken Ying-Kai Liao, Fuu-Jen Tsai

**Affiliations:** a Department of Medical Research, China Medical University Hospital, Taichung, Taiwan; b Artificial Intelligence Center, China Medical University Hospital, Taiwan; c Department of Medical Genetics, China Medical University Hospital, Taichung, Taiwan

**Keywords:** Artificial intelligence, Bone age assessment, Deep learning

## Abstract

**Introduction:**

A deep learning-based automatic bone age identification system (ABAIs) was introduced in medical imaging. This ABAIs enhanced accurate, consistent, and timely clinical diagnostics and enlightened research fields of deep learning and artificial intelligence (AI) in medical imaging.

**Aim:**

The goal of this study was to use the Deep Neural Network (DNN) model to assess bone age in months based on a database of pediatric left-hand radiographs.

**Methods:**

The Inception Resnet V2 model with a Global Average Pooling layer to connect to a single fully connected layer with one neuron using the Rectified Linear Unit (ReLU) activation function consisted of the DNN model for bone age assessment (BAA) in this study. The medical data in each case contained posterior view of X-ray image of left hand, information of age, gender and weight, and clinical skeletal bone assessment.

**Results:**

A database consisting of 8,061 hand radiographs with their gender and age (0–18 years) as the reference standard was used. The DNN model’s accuracies on the testing set were 77.4%, 95.3%, 99.1% and 99.7% within 0.5, 1, 1.5 and 2 years of the ground truth respectively. The MAE for the study subjects was 0.33 and 0.25 year for male and female models, respectively.

**Conclusion:**

In this study, Inception Resnet V2 model was used for automatic interpretation of bone age. The convolutional neural network based on feature extraction has good performance in the bone age regression model, and further improves the accuracy and efficiency of image-based bone age evaluation. This system helps to greatly reduce the burden on clinical personnel.

## 1. Introduction

Bone age assessment (BAA), or skeletal age evaluation, is a clinical method for analyzing the stage of skeletal maturation of children. BAA is performed usually by comparing an X-ray of non-dominant wrist with an atlas of known sample bones [1]. The famous manual methods Greulich and Pyle (GP method) [2,3] and Tanner-Whitehouse (TW method) [4] of BAA are time-consuming and rely on the experiences of attending physicians, and thus are prone to observer variability. The bone age can be used to evaluate the individual maturity precisely, and also can be the diagnosis reference of pediatric endocrine disorder. The regular process of bone age assessment in the hospital is using low dose X-ray from the subject's non-dominant hand [5].

Automatic bone age interpretation has long been a goal of radiology research. Currently most of the methods need to segment specific skeletons, as a region of interest (ROI), through manual or computer algorithms, and classification or regression is then applied. Seok et al. [6] used scale invariant feature transformation (SIFT) to extract image features and singular value decomposition (SVD) to create a fixed-dimensional eigenvector and applied a fully connected neural network to build models. Because of the limited number of images used, their model was inaccurate in the image analysis due to the huge difference in the internal data set, and it did not provide any quantifiable performance criteria. Somkantha et al. [7] projected carpal region on the horizontal and vertical axes to extract the boundary and morphological features of the wrist and used the support vector machine (SVM) to establish a model. Zhang et al. [8] extracted the characteristics from the carpal region and then used the fuzzy logic classifier for skeletal age assessment. However, these methods are less meaningful for children older than 7 years because the wrist of a 5 to 7-year-old child is usually mature [9]. Bone-Xpert is a successful system for the automated determination of skeletal age [10]. The system uses the active appearance model (AAM) to automatically segment the bone area of the hand and wrist. GP and TW2 bone ages are then calculated based on the bone shape, the brightness and texture characteristics. The standard deviation of the resulted skeletal ages is between 0.42 and 0.80 years. This system is the only equipment that is approved in Europe in this field. Also, it is the first commercial automated BAA software. Recently, artificial intelligence (AI) development in applying deep learning technology to a large number of images to train a neural network model is rapid. Hyunkwang Lee used around 10 thousand bone age images, resulted in an accuracy of 90% cases within 1 year. This is one of the most accurate and effective methods for the current AI-assisted interpretation of bone age [11].

In this study, an Inception-Resnet-v2 Neural Network as the base model was introduced. With training data of children and teenagers aged from 2 to 18 in Taiwan, the network can predict well when given only the non-dominant hand bone X-ray and the gender information. When a patient's medical image is input into the automatic bone age identification system (ABAIs), the image is automatically sent to the AI server and analyzed. The results are subsequently looped back to the ABAIs, which can provide a second opinion to assist the physician in diagnosis. This ABAIs is expected to reduce the interpretation error, and to actually reduce the complexity, time and cost of the process of skeletal age assessment.

## 2. Aim

The goal of this study was to use the Deep Neural Network (DNN) model to assess bone age in months based on a database of pediatric left-hand radiographs.

## 3. Methods

### 3.1. Clinical data collection

The study followed the protocol approved by the IRB of China Medical University Hospital and retrospectively collected medical data including 9,717 cases (CMUH107-REC2-097).

In this study, the left-hand X-ray generated in the conventional medical process was connected by the PACS (Picture Archiving and Communication System, medical image capture and transmission system) and finally received DICOM file (Digital Imaging and Communications in Medicine).

The DICOM files were already encrypted and de-identified to ensure the integrity, verification and confidentiality before released.

The medical data in each case contained posterior view of X-ray image of left hand, information of age, gender and weight, and clinical skeletal bone assessment. Cases of ages between 0 and 20 years old with their clinical records were selected and grouped by sex. Diagnostic results and clinical bone age assessment values by clinicians were used as references for the trained machine in deep learning interpretation and prediction of related symptoms.

### 3.2. Image labeling

Five professional pediatricians or radiologists with at least 5 years of experience in bone age assessment will perform bone age assessment based on the study subject's left-hand X-ray images collected in this trial.

In order to prevent the result of the system interpretation affecting the judgment of the clinician or the clinician discussed the case with each other, the subject's hand bone X-ray is given to a professional physician before all five physicians confirm the individual interpretation result. The cases cannot be discussed with each other, and take the median as the final labeling result. Moreover, the internal consistency between physicians have been estimated by intra-class correlation coefficient (ICC), ICC = 0.82 (0.65–0.89), p < 0.001.

### 3.3. Normalization

Due to the difference in hand size among different ages, proper field of view (FOV) was given to each case when the X-ray image was taken to follow the as low as reasonably achievable (ALARA) principle. As a result, in addition to the dimension differences of the X-ray images, the exposure index (EI) was different among the cases. To eliminate such differences, all the images were resized to the size of 256 × 256 pixels, and the image intensities were normalized to 0 ~ 255 gray-scale distribution. Saliency maps were generated based on the image features extracted from the normalized images by the AI system for BAA.

### 3.4. Deep neural network (DNN)

In the past, some researchers used the Inception V3 network as a model for medical image recognition. Although it performed well, the network that needed to be built was mass and complicated [12–13]. In general, the Inception network focuses on network depth, while Resnet focuses on network width. Combining the advantages of the two structures, we use Inception Resnet V2 and greatly improve the training efficiency [14]. The DNN model is based on the Inception Resnet V2 model with a Global Average Pooling layer to connect to a single fully connected layer with one neuron using the Rectified Linear Unit (ReLU) activation function, which represents the bone age of the subjects. The model was trained using the Adadelta optimization algorithm, which achieves faster convergence than most optimization algorithms. The model optimized the mean squared error (MSE) between the predicted bone age and the target bone age. The Inception Resnet V2 portion of the model was initialized using the weights of an Inception Resnet V2 model that was pre-trained on ImageNet database, while the fully connected layer was initialized using the initialization with uniform distribution. The model pipeline was shown in Supplementary 2.

The software was implemented using Python (version 3.6.0) through the Jupyter Interactive Notebook for fast prototyping. The model was built and trained using the Keras framework (version 2.1.6) with the Tensorflow framework (version 1.7) as the backend to Keras.

### 3.5. Statistical analysis

#### 3.5.1. Model performance

Since BAA is a regression problem, the statistical analysis techniques were limited to the MSE and mean absolute error (MAE). In order to carry out “accuracy” with a single predicted value, the absolute error with a threshold value was converted to a binary classification. This allows the scores, such as accuracy, precision and recall within 6 months, 1 year, 1.5 year and 2 years, to be obtained in this system.

In addition to evaluating the performance of the model on the test set and avoiding overfitting, the performance of 5-fold cross-validation is also evaluated.

The statistical analysis was also performed in Python. Lin's concordance correlation coefficient (CCC) [15] values were calculated to check the agreement in this study.

## 4. Results

### 4.1. The dataset

The cases of bone age assessments made from April 2003 to November 2017 were applied in the deep learning training. ABAIs conducted a retrospective and multicenter study with the primary endpoint to evaluate the software's performance in identifying X-ray images of non-dominant hand bone containing 9,717 cases from 16 clinical sites of CMUH (China Medical University Hospital) system in Taiwan. Patients who have duplicate image or poor image quality were excluded in this study (n = 1,656).

A retrospective training data, including 2,757 males and 4,454 females (total 7,211, Table 1), were selected to train the incV2resNet (Inception V2 Residual Net). After the completion of the training, 321 males and 529 females (total 850, Table 1) were used to test the model. In the testing stage, those with age younger than 2 years or older than 18 years were excluded, and the final testing data included 312 males and 508 females (total 820). Supplementary 1 shows the study flowchart.

### 4.2. Performance of data

We defined 4 different accuracies of difference to validate our model performance. Each represents the difference between AI predicted bone age and bone age assessment by the doctor. Table 2 lists the accuracies of AI predicted bone age and bone age assessment by the doctor. 99.7% of cases have the difference of less than 2 years (precision: 99.4%, recall: 100%). And within 2 years, the accuracy of 5-fold cross-validation was 97.9% (precision: 97.2%; recall rate: 99.0%).

In an agreement study, a concordance correlation coefficient (CCC) is usually used to measure how much agreement is. The CCC value in our study was 0.991 (with 0.990 and 0.992 as 95% confidence interval), which was considered almost perfect concordance [16]. AI predicted and doctor assessment bone age of Q-Q plot, residual and absolute difference distrubution are shown in Supplementary 4–6. Mean absolute error of total cases was 0.281 year (3.37 months) and mean square error of total cases was 0.203 year (2.4 months). Likewise, using 5-fold cross-validation to evaluate the performance of the model, MAE and MSE are 0.311 and 0.432 respectively (as shown in Table 2).

### 4.3. Display of ABAIs

**Fig. 1:**
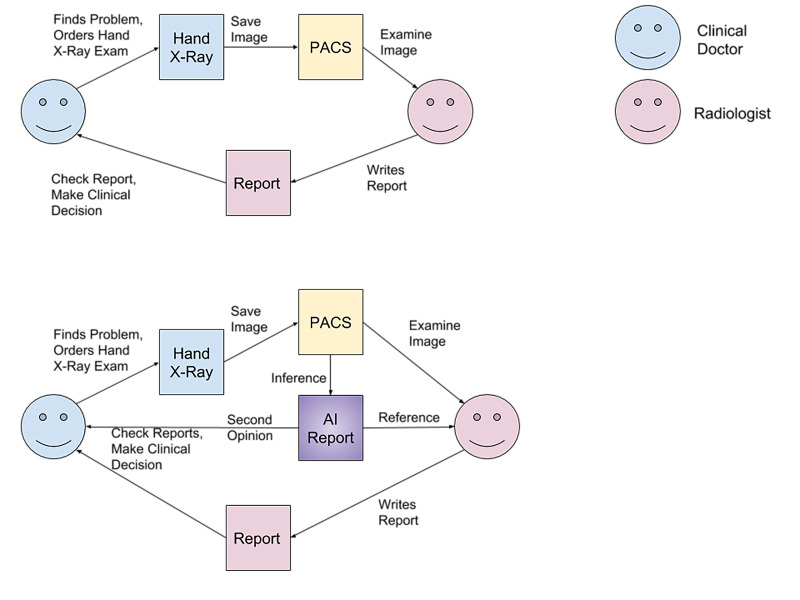
BAA work flow diagram of Traditional (Top) and AI assisted processes (Bottom).

Fig. 1 shows the difference between the AI intervention and the traditional process. In the traditional process, after the hand bone X-ray is taken, the image is sent to the radiologist who accordingly writes the report of the hand bone X-ray, and then the report is sent to the clinical doctor. The major difference between the two processes is the extra route of AI intervention. That is, after the hand bone X-ray is taken, ABAIs receives the hand bone X-ray image and outputs another report to the clinician as the second opinion.

**Fig. 2:**
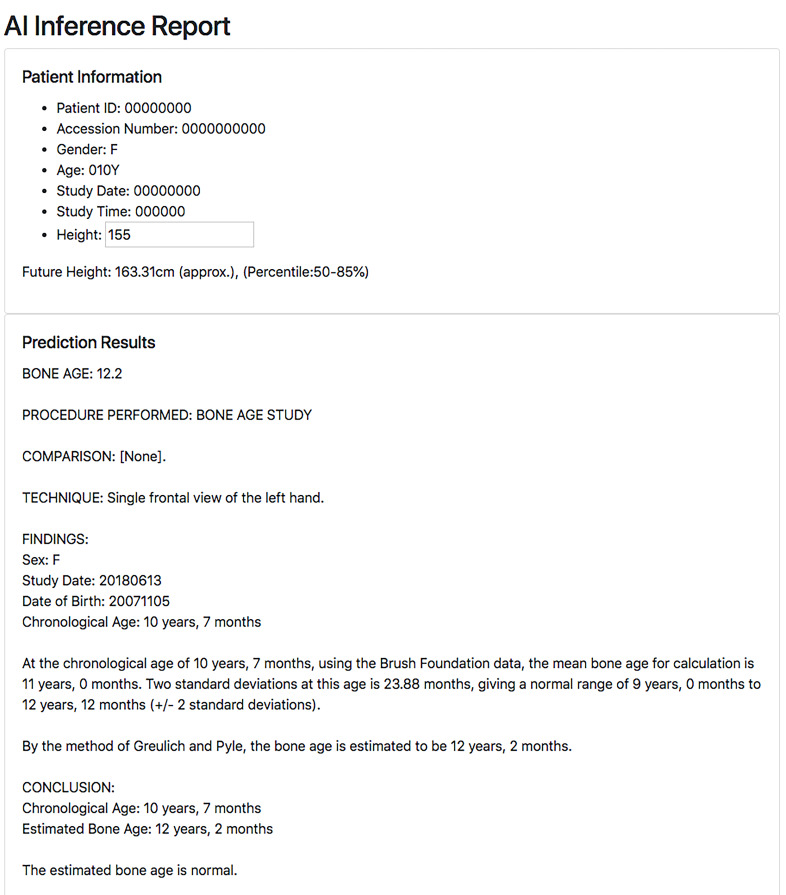
Report generated by the AI assisted AIBAAs with sensitive information replaced by 0's.

Fig. 2 shows a sample BAA report generated by the AI based system. The BAA values were acquired based on the saliency maps generated by the AI based system. For the usability, the first topic “Patient Information” includes the patient's personal information to make sure the specific BAA report is for the right patient. The second topic “Prediction Results” shows the AI-predicted bone age. The standard deviation of the patient makes reference to the standard deviation table from Brush Foundation data in reference [9]. The age range is calculated by using the mean and standard deviation and it would be conclude that “The estimated bone age is normal.” if the patient's bone age is within the age range.

To clarify the position that ABAIs utilized to determine the bone age, Supplementary 3 shows a sample saliency map generated by the AI system for BAA. In deep learning fields, a saliency map can always help to check whether the neural network focuses on the right parts of images. Supplementary 3 shows that the ABAIs focuses on carpal and metacarpal bones which exactly follow the rule in the guidance book of hand bone age [9]. It is also proved that ABAIs has captured the correct and important information from the X-ray images.

## 5. Discussion

For the past 20 years, assessing bone age has been a very tedious, repetitive and time-consuming task. Different physicians may have various assessment results for the same radiograph. Therefore, increasing accuracy and efficiency is very important.

In recent years, the field of computer vision has been developing rapidly. Different algorithms have been improved, and predictions are consequently faster and more accurate. However, machine learning relies on the quality and quantity of data in predicting accuracy. With high-quality and high-volume imagery, machine learning can be well suited to applications in the medical field.

The previous study conducted by the team at MGH excluded subjects with bone age between 0 and 4 years old, while in this study such cases were included. And it is still demonstrated that the AI system is able to achieve a better score in general [11]. The previous study also used a softmax function for the output, whereas ReLU function was used in this study in order to allow the model to be as precise as possible in the output. With such improvement, the model in this study achieved a better MAE score, 94.0% for the male model and 96.1% for the female model with regard to the “within 1 year” accuracy, which is significantly better than the previous study. Furthermore, the preprocessing applied in this study was much faster than the previous one. The preprocessing in this study was a simple pad and resizing, while in the previous study it included segmentation in addition to resizing. For each image, it takes about 4 ms to process on an NVIDIA GPU computer, which is an upgrade from the 10 ms processing and 1 s segmentation time required in the previous study.

In each BAA, the radiologist compares the client's X-ray image with GP reference images. Not only the process is tedious and time-consuming, but also the results of the assessment are quite different among clinicians because the bone age is evaluated on an individual basis. Therefore, one of the major advantages of the automatic AI system for skeletal age assessment is consistency that removes the differences in inspection due to different readers. The fully automated deep learning system was built so that the BAA results can be automatically obtained after the images are input to CNN. Moreover, a prediction of the final height after cessation of growth is prepared as well by the AI system simultaneously within the structured radiology report. These results are finally interpreted and reported by the radiologists. This system accepts images from different hospitals, instruments and different radiologists. After automatic normalization of the images, the accuracy of the skeletal BAA for men and women achieves 95% or above. With automatically generated BAA results and displayed three to five GP reference images, the radiologist just needs a click of mouse and a structured BAA radiology report is generated in which the assessed skeletal age and possible future height are described.

Although the BAA results by BoneXpert have been fairly accurate, there are still several potential problems with the system. BoneXpert only uses 1,559 hand X-ray images in its system and those images are all from European and American people. As a consequence, there may be possible misjudgment when it is applied to the Asian race. In an earlier study, BoneXpert was applied to 397 children's hand bone images, but up to 139 cases were wrongly evaluated [15]. Another disadvantage of BoneXpert is that the wrist bones of young children, which possibly contain significant image information, are not used in the skeletal BAA.

Recently, Radiological Society of North America (RSNA) hosted the skeletal age prediction contest, 2017 Pediatric Bone Age Challenge, for which the host revealed 12,000 images of the European and American people to the participants. Iglovikov *et al.* used this dataset to train the neural network model and tested BAA for both sexes and different ages. The accuracy of the skeletal BAA still had room for improvement. Besides, no prediction was made for the final height of the clients [17].

Recent studies have shown automatic bone age assessment for all age ranges, races and genders [18]. And the bone age assessment based on deep learning and Gaussian process regression has achieved great success [19]. However, many studies have pointed out that the influence of different ethnic groups can affect bone development and assessment of bone age [20–21]. Therefore, further verification based on Asian ethnic groups is still necessary. Because of the differences in genes and lifestyle habits, the BAA systems developed on the basis of European and American races may not necessarily be suitable for the Asian race. Therefore, this AI system is applied to the hand X-ray images of Asian people for automatic BAA with deep learning technology. Nevertheless, the AI system still has many challenges to overcome such as patient privacy, imaging that requires professional physicians to interpret, human anatomical differences and different clinical manifestations of the same disease. These are all problems that are not found in general images. Medical images are particularly challenging because the specific domain knowledge is required to interpret these images. However, with the enhanced neural network which is now widely applied, the AI system can increase the BAA efficiency and accuracy. In addition, the future height of the client is automatically predicted in a structured report for clinical use.

We have already presented the performance report of the model in this study. However, this article did not mention the application of bone age assessment in other hospitals or cities in Taiwan, more clinical trials will need to be conducted to prove model effectiveness in the future.

ABAIs has successfully achieved the following goals: (a) demonstrated the application of ML and AI in medical imaging; (b) tools and methods were used to stimulate the field of ML to help solve other diagnostic problems; (c) it provides more accurate, efficient, and timely results of bone age diagnosis, and can be applied for clinical teaching in hospitals, thus reducing the workload of physicians and provides physician-assisted diagnoses.

## Supplementary Data

Supplementary Fig. 1 Flowchart of radiographs enrolled in the study.

Supplementary Fig. 2 Schematic diagram of ABAIs.

Supplementary Fig. 3 An example of saliencymap (right), showing the significant features learned froma clinical image (left), by deep learning for ABAIs.

Supplementary Fig. 4 The Q-Q plot of AI predicted bone age (y-axis) and doctor assessment bone age (x-axis).

Supplementary Fig. 5 The difference between AI predicted bone age and doctor assessment bone age.

Supplementary Fig. 6 The sorting absolute difference of years between AI predicted bone age and doctor assessment bone age.

## Figures and Tables

**Table 1:** The age distribution of the dataset images by training set and testing set.

Age (years)	Training set			Testing set		
	
	Male	Female	Total	Male	Female	Total
**Total**	**2,757**	**4,454**	**7,211**	**321**	**529**	**850**
0–2	3	15	18	9	20	29
2–3	5	18	23	4	8	12
3–4	22	24	46	11	7	18
4–5	35	32	67	10	14	24
5–6	49	58	107	13	15	28
6–7	57	156	213	16	45	61
7–8	66	322	388	17	74	91
8–9	111	593	704	9	106	115
9–10	122	624	746	27	84	111
10–11	229	673	902	33	56	89
11–12	380	545	925	50	33	83
12–13	415	488	903	39	26	65
13–14	363	375	738	28	21	49
14–15	315	305	620	27	8	35
15–16	290	126	416	14	9	23
16–17	166	63	229	7	1	8
17–18	90	28	118	7	1	8
18–20	39	9	48	0	1	1

**Table 2:** The system performance in testing data and 5-fold cross validation.

	Testing set			5-fold cross validation		
	
	Total	Male	Female	Total	Male	Female
					
	N = 787	N = 298	N = 489	N = 1,442	N = 551	N = 891
**Accuracy**						
<0.5 year	0.774	0.728	0.802	0.722	0.714	0.735
<1.0 year	0.953	0.940	0.961	0.911	0.903	0.922
<1.5 year	0.991	0.990	0.992	0.977	0.974	0.981
<2.0 year	0.997	1.000	0.996	0.979	0.975	0.984
**Precision**						
<0.5 year	0.781	0.742	0.807	0.716	0.712	0.724
<1.0 year	0.952	0.943	0.960	0.882	0.879	0.903
<1.5 year	0.986	0.984	0.986	0.967	0.963	0.969
<2.0 year	0.994	1.000	0.992	0.972	0.964	0.972
**Recall**						
<0.5 year	0.751	0.699	0.795	0.760	0.718	0.779
<1.0 year	0.955	0.938	0.962	0.946	0.909	0.960
<1.5 year	0.997	0.997	1.000	0.987	0.983	0.993
<2.0 year	1.000	1.000	1.000	0.990	0.987	0.997
**Mean Absolute error (year)**	**0.281**	**0.332**	**0.250**	**0.311**	**0.365**	**0.285**
**Mean Square error (year)**	**0.203**	**0.236**	**0.183**	**0.432**	**0.452**	**0.409**
