# Supplementary figures and images for: Report of clinical bone age assessment using deep learning for an Asian population in Taiwan
Source: Biomedicine (Taipei). 2021 Sep 1;11(3):50–8. doi: 10.37796/2211-8039.1256 (PMC8823497; doi:10.37796/2211-8039.1256)

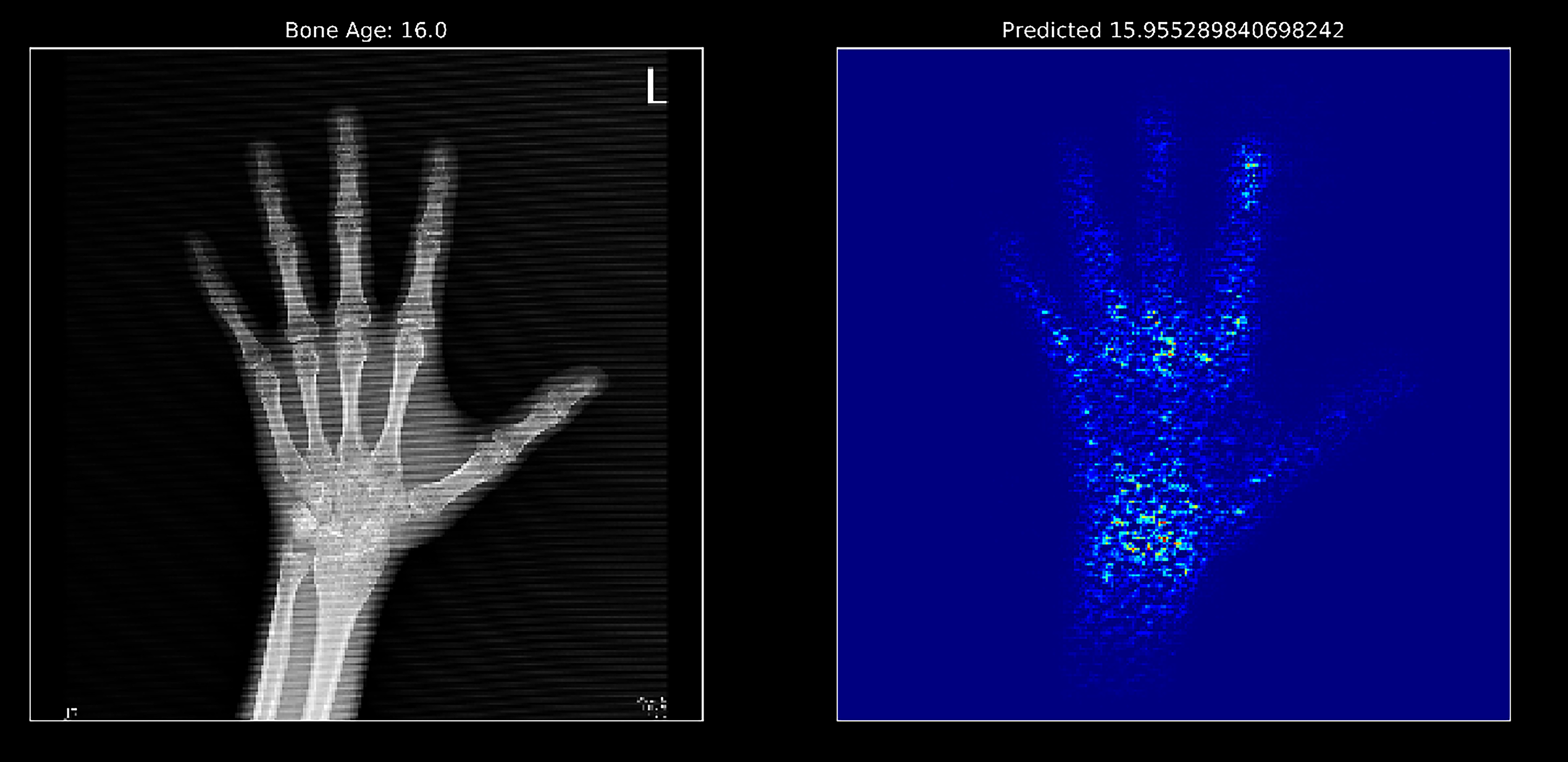

Supplement: Supplementary Fig. 3 — An example of saliencymap (right), showing the significant features learned froma clinical image (left), by deep learning for ABAIs. [file bmed-11-03-050-g005.tif]

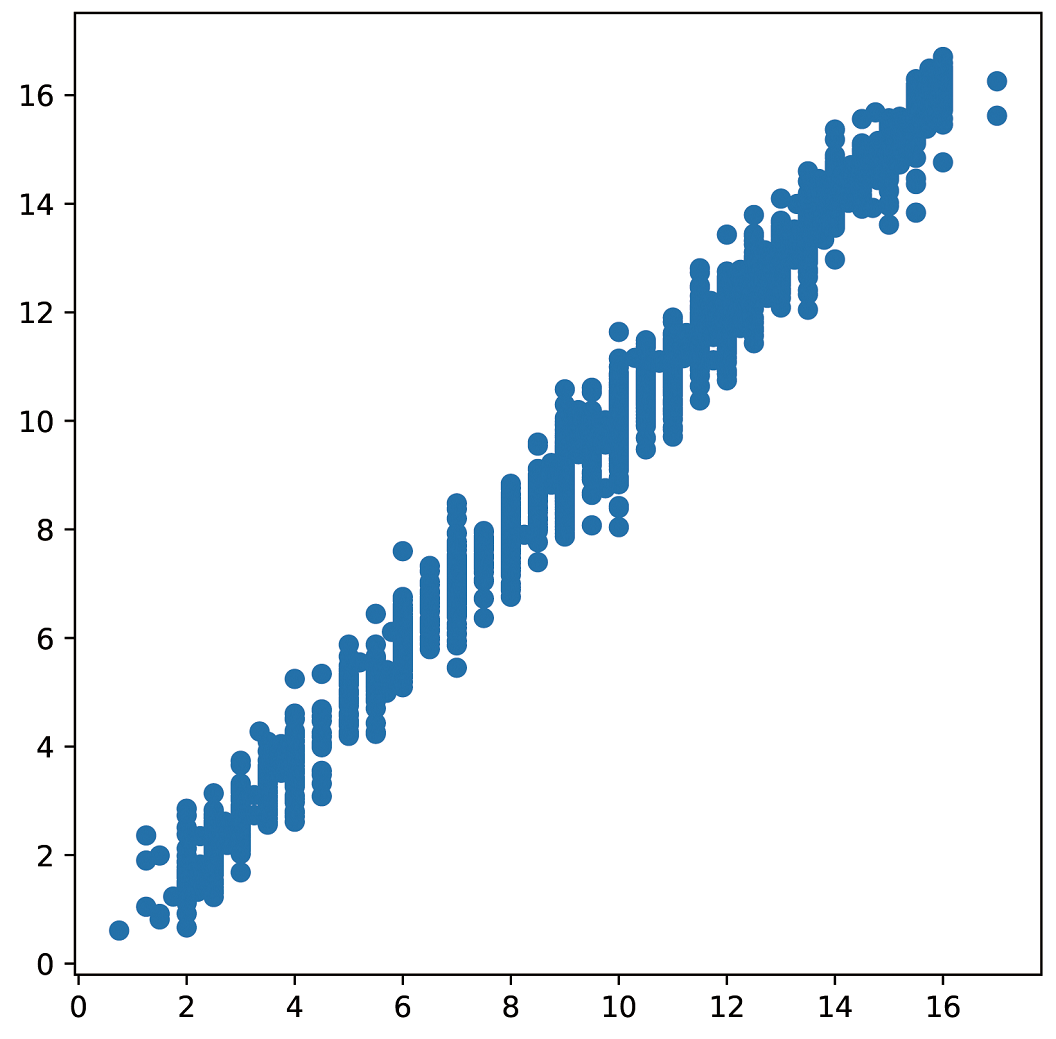

Supplement: Supplementary Fig. 4 — The Q-Q plot of AI predicted bone age (y-axis) and doctor assessment bone age (x-axis). [file bmed-11-03-050-g006.tif]

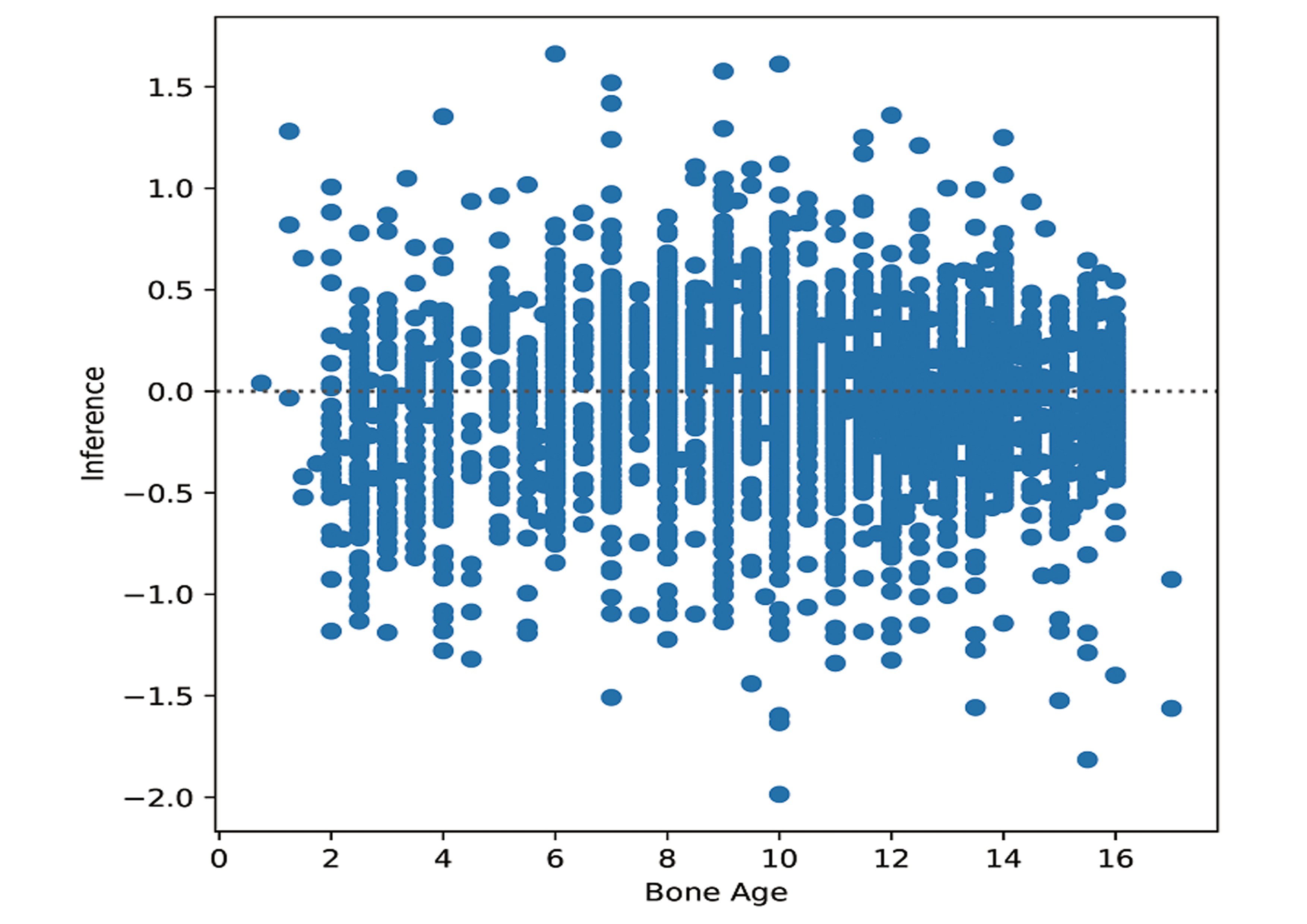

Supplement: Supplementary Fig. 5 — The difference between AI predicted bone age and doctor assessment bone age. [file bmed-11-03-050-g007.tif]

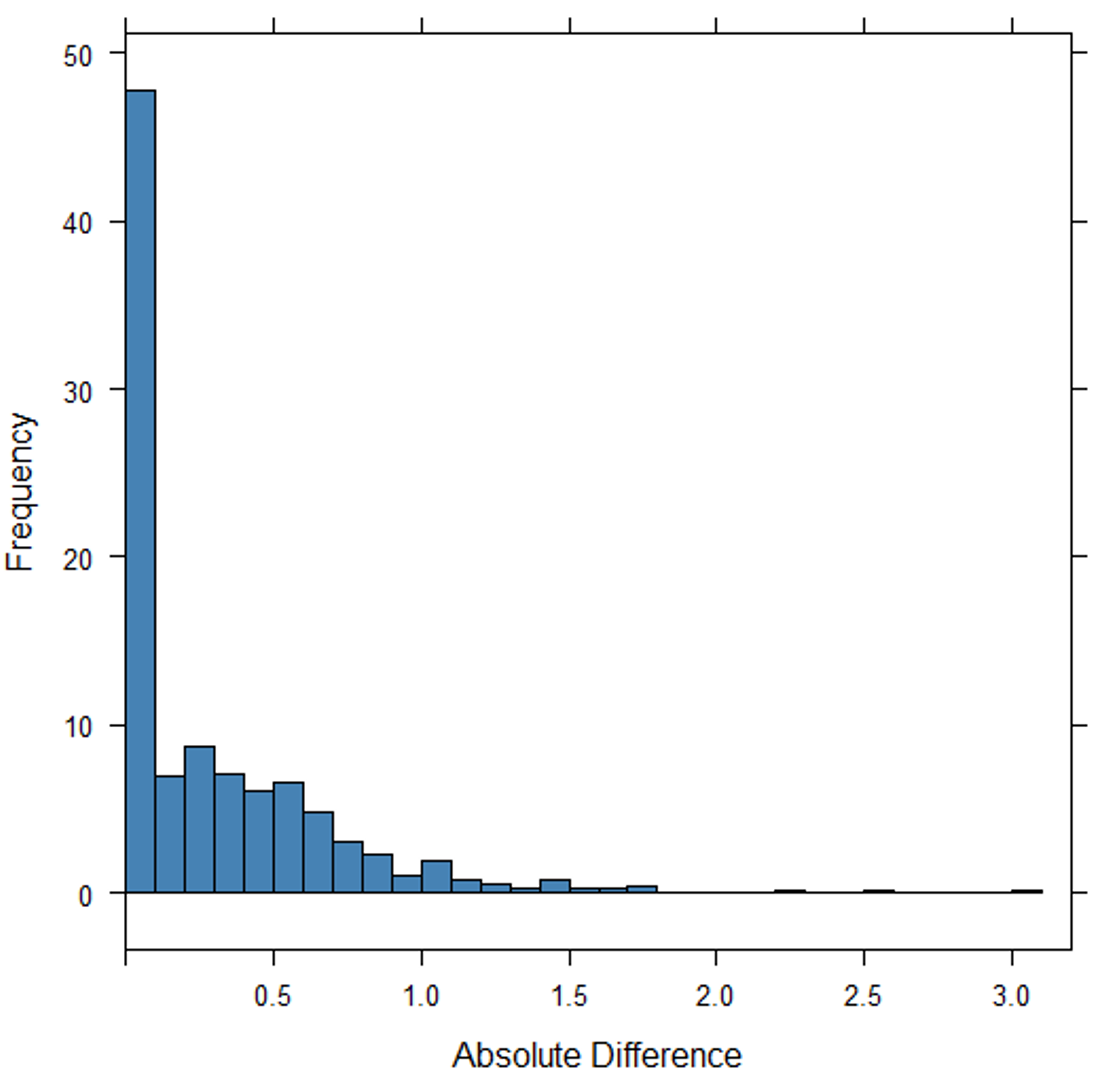

Supplement: Supplementary Fig. 6 — The sorting absolute difference of years between AI predicted bone age and doctor assessment bone age. [file bmed-11-03-050-g008.tif]
