# Supplementary material for: Report of clinical bone age assessment using deep learning for an Asian population in Taiwan
Source: Biomedicine (Taipei). 2021 Sep 1;11(3):50–8. doi: 10.37796/2211-8039.1256 (PMC8823497; doi:10.37796/2211-8039.1256)
Supplement: Supplementary file 7 [file bmed-11-03-050-s001.docx]

Comments from the reviewer:

The authors developed a deep learning model to identify bone age. The model utilizedInception Resnet V2 model with a Global Average Pooling layer to connect to a single fully connected layer with one neuron using the Rectified Linear Unit(ReLU) activation. The model’s accuracies on the testing set were 77.4%, 95.3%,99.1%, and 99.7% within 0.5, 1, 1.5, and 2 years of the ground truth, respectively. The MAE for the study subjects was 0.33 and 0.25 years for male and female models, respectively.The manuscript is well structured, and the procedures are logically sound. However, I do have some questions with regards to the manuscript:

1. The dataset was labeled by five experts. What is the consistency of the labels？

**Ans:**

We have conducted intra-class correlation coefficient, ICC is 0.82 (0.65-0.89), p<0.001 and we have added it in the manuscript. Thank you for the reminder of the reviewer.

1. The authors claimed that the proposed model (Inception Resnet V2 with ReLU) demonstrated good performance. What’s the explanation for combing these two methods?

**Ans:**

Thank you for this question. Inception network focuses on network depth, while Resnet focuses on network width. In fact, Inception Resnet V2 does not improve much accuracy compared to Inception V4 network, but it can improve training efficiency [1]. We have added more descriptions in the manuscript.

1. What's the novelty of this work?

**Ans:**

This work proposes an Inception ResNet V2 which through fine-tuning and data augmentation allows to assess x-ray images of bone age.

The network learns the relevant features for regression which becomes particularly important since it is not always easy to distinguish which features should be extracted using traditional methods. Moreover, this is the first study using Inception ResNet V2 model to apply to the Taiwanese population.

1. Can the proposed model be used in images from other hospitals

**Ans:**

Although this article has not mentioned the application of Bone age assessment in other hospitals or cities, we are currently conducting clinical trials to apply this model in different hospitals. We have added more descriptions in the discussion part.
