# Supplementary material for: Report of clinical bone age assessment using deep learning for an Asian population in Taiwan
Source: Biomedicine (Taipei). 2021 Sep 1;11(3):50–8. doi: 10.37796/2211-8039.1256 (PMC8823497; doi:10.37796/2211-8039.1256)
Supplement: Supplementary file 8 [file bmed-11-03-050-s002.docx]

| **Table 1. The age distribution of the dataset images by training set and testing set.** | | | | | | | |
| --- | --- | --- | --- | --- | --- | --- | --- |
|  | **Training set** | | |  | **Testing set** | | |
| **Age (years)** | **Male** | **Female** | **Total** |  | **Male** | **Female** | **Total** |
| **Total** | 2,757 | 4,454 | 7,211 |  | 321 | 529 | 850 |
| 0-2 | 3 | 15 | 18 |  | 9 | 20 | 29 |
| 2-3 | 5 | 18 | 23 |  | 4 | 8 | 12 |
| 3-4 | 22 | 24 | 46 |  | 11 | 7 | 18 |
| 4-5 | 35 | 32 | 67 |  | 10 | 14 | 24 |
| 5-6 | 49 | 58 | 107 |  | 13 | 15 | 28 |
| 6-7 | 57 | 156 | 213 |  | 16 | 45 | 61 |
| 7-8 | 66 | 322 | 388 |  | 17 | 74 | 91 |
| 8-9 | 111 | 593 | 704 |  | 9 | 106 | 115 |
| 9-10 | 122 | 624 | 746 |  | 27 | 84 | 111 |
| 10-11 | 229 | 673 | 902 |  | 33 | 56 | 89 |
| 11-12 | 380 | 545 | 925 |  | 50 | 33 | 83 |
| 12-13 | 415 | 488 | 903 |  | 39 | 26 | 65 |
| 13-14 | 363 | 375 | 738 |  | 28 | 21 | 49 |
| 14-15 | 315 | 305 | 620 |  | 27 | 8 | 35 |
| 15-16 | 290 | 126 | 416 |  | 14 | 9 | 23 |
| 16-17 | 166 | 63 | 229 |  | 7 | 1 | 8 |
| 17-18 | 90 | 28 | 118 |  | 7 | 1 | 8 |
| 18-20 | 39 | 9 | 48 |  | 0 | 1 | 1 |
