# Supplementary material for: Report of clinical bone age assessment using deep learning for an Asian population in Taiwan
Source: Biomedicine (Taipei). 2021 Sep 1;11(3):50–8. doi: 10.37796/2211-8039.1256 (PMC8823497; doi:10.37796/2211-8039.1256)
Supplement: Supplementary file 9 [file bmed-11-03-050-s003.docx]

| **Table 2. The system performance in testing data and 5-fold cross validation.** | | | | | | |
| --- | --- | --- | --- | --- | --- | --- |
|  | **Testing set** | | | **5-fold cross validation** | | |
|  | **Total** | **Male** | **Female** | **Total** | **Male** | **Female** |
|  | **N= 787** | **N= 298** | **N= 489** | **N = 1,442** | **N = 551** | **N = 891** |
| **Accuracy** |  |  |  |  |  |  |
| <0.5 year | 0.774 | 0.728 | 0.802 | 0.722 | 0.714 | 0.735 |
| <1.0 year | 0.953 | 0.940 | 0.961 | 0.911 | 0.903 | 0.922 |
| <1.5 year | 0.991 | 0.990 | 0.992 | 0.977 | 0.974 | 0.981 |
| <2.0 year | 0.997 | 1.000 | 0.996 | 0.979 | 0.975 | 0.984 |
| **Precision** |  |  |  |  |  |  |
| <0.5 year | 0.781 | 0.742 | 0.807 | 0.716 | 0.712 | 0.724 |
| <1.0 year | 0.952 | 0.943 | 0.960 | 0.882 | 0.879 | 0.903 |
| <1.5 year | 0.986 | 0.984 | 0.986 | 0.967 | 0.963 | 0.969 |
| <2.0 year | 0.994 | 1.000 | 0.992 | 0.972 | 0.964 | 0.972 |
| **Recall** |  |  |  |  |  |  |
| <0.5 year | 0.751 | 0.699 | 0.795 | 0.760 | 0.718 | 0.779 |
| <1.0 year | 0.955 | 0.938 | 0.962 | 0.946 | 0.909 | 0.960 |
| <1.5 year | 0.997 | 0.997 | 1.000 | 0.987 | 0.983 | 0.993 |
| <2.0 year | 1.000 | 1.000 | 1.000 | 0.990 | 0.987 | 0.997 |
| **Mean Absolute error (year)** | 0.281 | 0.332 | 0.250 | 0.311 | 0.365 | 0.285 |
| **Mean Square error (year)** | 0.203 | 0.236 | 0.183 | 0.432 | 0.452 | 0.409 |
