# Supplementary material for: Report of clinical bone age assessment using deep learning for an Asian population in Taiwan
Source: Biomedicine (Taipei). 2021 Sep 1;11(3):50–8. doi: 10.37796/2211-8039.1256 (PMC8823497; doi:10.37796/2211-8039.1256)
Supplement: Supplementary file 10 [file bmed-11-03-050-s004.docx]

**Report of Clinical Bone Age Assessment using Deep Learning for an Asian population in Taiwan**

Chi-Fung Cheng^1^, Eddie Tzung-Chi Huang^2,3^, Ken Ying-Kai Liao^2^, Jung-Tsung Kuo^2^,

Fuu-Jen Tsai^4,*^

^1^Department of Medical Research, China Medical University Hospital, Taichung, Taiwan

^2^Artificial Intelligence Center, China Medical University Hospital, Taiwan

^3^Department of Bioinformatics and Medical Engineering, Asia University, Taichung, Taiwan

^4^Department of Medical Genetics, China Medical University Hospital, Taichung, Taiwan

*Corresponding Author: Fuu-Jen Tsai, Email: d0704@mail.cmuh.org.tw
